# (*R*)-(+)-Dimeth­yl[4-oxido-2-oxo-1-(1-phenyl­eth­yl)-1,2,5,6-tetra­hydro­pyridin-3-yl]sulfonium

**DOI:** 10.1107/S1600536810052955

**Published:** 2011-01-08

**Authors:** Paola G. Gordillo, Joel L. Terán, Jorge R. Juárez, Angel Mendoza

**Affiliations:** a Centro de Química, ICUAP, Benemérita Universidad Autónoma de Puebla, 72570, Puebla, Pue., Mexico

## Abstract

In the title zwitterionic compound, C_15_H_19_NO_2_S, the six-membered heterocycle adopts a sofa conformation. The negative charge is delocalized along the carbonyl and enolate system on the ring and the positive charge is localized on the S atom. Two inter­molecular C—H⋯O inter­actions help to establish the packing.

## Related literature

For background to the synthesis of chiral non-racemic zwitterionic 4-alk­oxy-3-sulfonium ylide pyridine-2-ones, see: Zang *et al.* (2008 ▶); Kappe *et al.* (1983 ▶); Palillero *et al.* (2009 ▶). For the biological activity of related structures, see: Basco *et al.* (1994 ▶); Koruzňjak *et al.*, 2003 ▶). For ring conformation analysis, see: Cremer & Pople (1975 ▶).
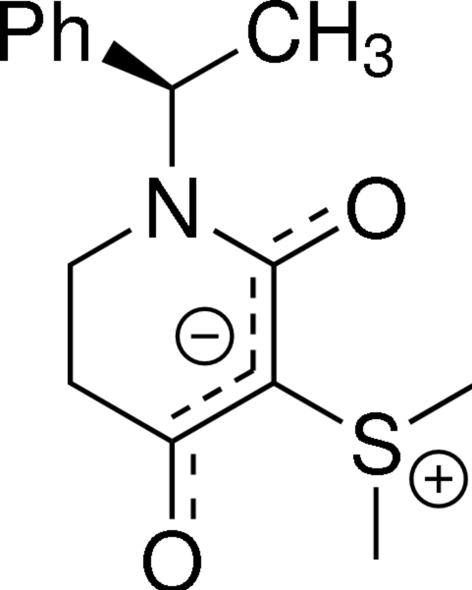


## Experimental

### 

#### Crystal data

C_15_H_19_NO_2_S
*M* *_r_* = 277.37
Orthorhombic, 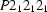

*a* = 5.9860 (17) Å
*b* = 7.4050 (14) Å
*c* = 31.589 (5) Å
*V* = 1400.2 (5) Å^3^
*Z* = 4
Mo *K*α radiation
μ = 0.23 mm^−1^
*T* = 293 K
0.5 × 0.4 × 0.2 mm

#### Data collection

Siemens P4 diffractometer
Absorption correction: ψ scan (North *et al.*, 1968 ▶) *T*_min_ = 0.728, *T*_max_ = 0.846
3016 measured reflections
2683 independent reflections
1928 reflections with *I* > 2σ(*I*)
*R*_int_ = 0.045
3 standard reflections every 97 reflections  intensity decay: 3%

#### Refinement

*R*[*F* ^2^ > 2σ(*F* ^2^)] = 0.061
*wR*(*F* ^2^) = 0.153
*S* = 1.03
2683 reflections
172 parameters
H-atom parameters constrained
Δρ_max_ = 0.63 e Å^−3^
Δρ_min_ = −0.39 e Å^−3^
Absolute structure: Flack (1983 ▶), 532 Friedel pairs
Flack parameter: −0.01 (16)

### 

Data collection: *XSCANS* (Siemens, 1994 ▶); cell refinement: *XSCANS*; data reduction: *XSCANS*; program(s) used to solve structure: *SIR2004* (Burla *et al.*, 2005 ▶); program(s) used to refine structure: *SHELXL97* (Sheldrick, 2008 ▶); molecular graphics: *ORTEP-3 for Windows* (Farrugia, 1997 ▶); software used to prepare material for publication: *WinGX* (Farrugia, 1999 ▶).

## Supplementary Material

Crystal structure: contains datablocks global, I. DOI: 10.1107/S1600536810052955/bt5438sup1.cif

Structure factors: contains datablocks I. DOI: 10.1107/S1600536810052955/bt5438Isup2.hkl

Additional supplementary materials:  crystallographic information; 3D view; checkCIF report

## Figures and Tables

**Table 1:** Hydrogen-bond geometry (Å, °)

*D*—H⋯*A*	*D*—H	H⋯*A*	*D*⋯*A*	*D*—H⋯*A*
C7—H7*C*⋯O2^i^	0.96	2.36	3.315 (6)	172
C15—H15*A*⋯O1^ii^	0.96	2.38	3.167 (5)	138

Symmetry codes: (i) 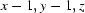; (ii) 
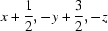
.

**Fig. 1.**
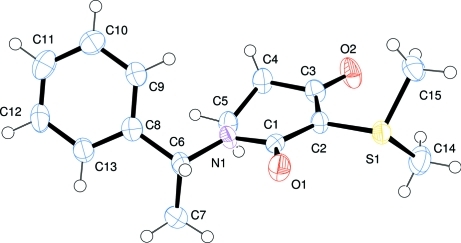
The molecular structure of the title compound with 50% probability displacement ellipsoids for non-H atoms.

## supplementary crystallographic information

### Comment

The synthesis of chiral non racemic zwitterionic 4-alkoxy-3-sulfonium ylide pyridine-2-ones is an original area of interest in organic chemistry (Zang *et al.*, 2008; Kappe *et al.*, 1983) because they are useful for the synthesis of piperidine-2,4-dione and pyridine-2-one (Palillero *et al.*, 2009) compounds and because of their interesting biological properties  (Basco *et al.*, 1994; Koruzňjak *et al.*, 2003).

The title compound I, features a zwitterionic molecule. The chiral centre  shows an *R* configuration with [α]_D_= +70.5. The six member ring N1/C1/C2/C3/C4/C5 shows an sofa conformation with puckering parameters (Cremer & Pople, 1975) *Q* = 0.465 (4) Å, θ_2_ = 119.7 (5)°, φ_2_ = 103.2 (6)°, q_2_ = 0.404 (4) Å and q_3_ = -0.231 (4) Å. The bond distances of N1—C1, N1—C5, C5—C4 and C4—C3 show typical values, so that C2—C3 distance shows a single double bond (1.415 (5) Å), while C1—C2 (1.455 (5) Å) distance is shorter than common *sp*^3^—sp^3^ bonds, furthermore C3—O2 (1.244 (5) Å) and C1—O1 (1.250 (4) Å) distances are longer than related enolates and amide groups respectively these values suggest that negative charge is delocalized over O1/C1/C2/C3/O2 system and in the sulfonium group is located the positive charge in the zwitterion. Crystal packing is stabilized by two weak intermolecular C —H···O interactions.

### Experimental

The title compound, was obtained by an intramolecular cyclization reaction of (1'*R*)-(+)-{[(2-methoxycarbonyl-ethyl)-(1'-phenyl-ethyl)-carbamoyl]-methyl}-dimethyl-sulfonium; bromide (1 mmol), which was dissolved in CH_3_CN (10 mL), treated with KOH (1.2 mmol) and stirred for two hours at room temperature. The resulting mixture was concentrated in vacuum and dissolved in ethyl acetate, filtered and concentrated giving the desired compound in 95%. Crystals were obtained from an ethyl acetate/diethylether solution; m.p. 139–140°C, [α]_D_= +70.5 (*c* 1.1, MeOH). IR (KBr) 1656 cm^-1. 1^H NMR (400 MHz, CDCl_3_) δ (p.p.m., *J* Hz): 1.51 (d, *J* = 7.2, 3H, Me), 2.32 (m, 2H), 2.90 (m, 1H), 2.98 (s, 3H, SMe), 3.00 (s, 3H, SMe), 3.16 (m, 1H), 5.94 (q, *J* =7.2, 1H), 7.27–7.40 (m, 5H). ^13^C NMR (100 MHz, CDCl_3_) δ p.p.m. 15.4, 26.0, 26.3, 36.3, 37.6, 48.9, 74.3, 126.5–141.0, 166.2, 187.5. HRMS (FAB): Calcd for C_15_H_19_NO_2_S: 277.1124. Found: 277.1103.

### Refinement

H atoms linked to C atoms were placed in geometrical idealized positions and refined as riding on their parent atoms, with C—H = 0.93–0.98 Å and with *U*_iso_(H) = 1.2 *U*_eq_(C) or *U*_eq_(H) = 1.5 *U*_eq_(C) for methyl groups.

### Crystal data

**Table:**

C_15_H_19_NO_2_S	*F*(000) = 592
*M**_r_* = 277.37	*D*_x_ = 1.316 Mg m^−^^3^
Orthorhombic, *P*2_1_2_1_2_1_	Mo *K*α radiation, λ = 0.71073 Å
Hall symbol: P 2ac 2ab	Cell parameters from 46 reflections
*a* = 5.9860 (17) Å	θ = 21.3–35.1°
*b* = 7.4050 (14) Å	µ = 0.23 mm^−^^1^
*c* = 31.589 (5) Å	*T* = 293 K
*V* = 1400.2 (5) Å^3^	Prism, colorless
*Z* = 4	0.5 × 0.4 × 0.2 mm

### Data collection

**Table:**

Siemens P4 diffractometer	*R*_int_ = 0.045
graphite	θ_max_ = 29.0°, θ_min_ = 2.6°
2θ/ω scans	*h* = −1→8
Absorption correction: ψ scan (North *et al.*, 1968)	*k* = −1→10
*T*_min_ = 0.728, *T*_max_ = 0.846	*l* = −43→1
3016 measured reflections	3 standard reflections every 97 reflections
2683 independent reflections	intensity decay: 3%
1928 reflections with *I* > 2σ(*I*)	

### Refinement

**Table:**

Refinement on *F*^2^	Secondary atom site location: difference Fourier map
Least-squares matrix: full	Hydrogen site location: inferred from neighbouring sites
*R*[*F*^2^ > 2σ(*F*^2^)] = 0.061	H-atom parameters constrained
*wR*(*F*^2^) = 0.153	*w* = 1/[σ^2^(*F*_o_^2^) + (0.0631*P*)^2^ + 1.1965*P*] where *P* = (*F*_o_^2^ + 2*F*_c_^2^)/3
*S* = 1.03	(Δ/σ)_max_ < 0.001
2683 reflections	Δρ_max_ = 0.63 e Å^−^^3^
172 parameters	Δρ_min_ = −0.39 e Å^−^^3^
0 restraints	Absolute structure: Flack (1983), 532 Friedel pairs
Primary atom site location: structure-invariant direct methods	Flack parameter: −0.01 (16)

### Special details

**Table:**

Geometry. All s.u.'s (except the s.u. in the dihedral angle between two l.s. planes) are estimated using the full covariance matrix. The cell s.u.'s are taken into account individually in the estimation of s.u.'s in distances, angles and torsion angles; correlations between s.u.'s in cell parameters are only used when they are defined by crystal symmetry. An approximate (isotropic) treatment of cell s.u.'s is used for estimating s.u.'s involving l.s. planes.
Refinement. Refinement of *F*^2^ against ALL reflections. The weighted *R*-factor *wR* and goodness of fit *S* are based on *F*^2^, conventional *R*-factors *R* are based on *F*, with *F* set to zero for negative *F*^2^. The threshold expression of *F*^2^ > 2σ(*F*^2^) is used only for calculating *R*-factors(gt) *etc*. and is not relevant to the choice of reflections for refinement. *R*-factors based on *F*^2^ are statistically about twice as large as those based on *F*, and *R*- factors based on ALL data will be even larger.

### Fractional atomic coordinates and isotropic or equivalent isotropic displacement parameters (Å^2^)

**Table:**

	*x*	*y*	*z*	*U*_iso_*/*U*_eq_	
S1	1.10027 (19)	0.87732 (14)	0.03372 (3)	0.0323 (2)	
O1	0.8859 (6)	0.5383 (4)	0.05505 (8)	0.0393 (7)	
N1	0.7630 (6)	0.6122 (4)	0.12114 (10)	0.0300 (7)	
O2	1.0851 (7)	1.1013 (4)	0.11356 (10)	0.0511 (9)	
C9	0.8558 (7)	0.3759 (6)	0.19114 (12)	0.0351 (9)	
H9	0.9723	0.4434	0.1798	0.042*	
C1	0.8724 (7)	0.6511 (5)	0.08437 (10)	0.0278 (8)	
C6	0.6341 (8)	0.4423 (5)	0.12418 (12)	0.0345 (10)	
H6	0.7011	0.3572	0.1041	0.041*	
C4	0.8971 (9)	0.8843 (6)	0.15731 (11)	0.0373 (9)	
H4A	1.0124	0.8199	0.1727	0.045*	
H4B	0.8501	0.9863	0.1744	0.045*	
C3	0.9911 (8)	0.9518 (6)	0.11576 (12)	0.0340 (9)	
C8	0.6557 (7)	0.3579 (5)	0.16847 (12)	0.0305 (9)	
C10	0.8816 (9)	0.2937 (6)	0.23044 (13)	0.0404 (10)	
H10	1.0143	0.3077	0.2454	0.049*	
C15	1.3821 (8)	0.9447 (7)	0.04512 (13)	0.0427 (11)	
H15A	1.4586	0.9711	0.0191	0.064*	
H15B	1.4578	0.8487	0.0596	0.064*	
H15C	1.3805	1.0505	0.0627	0.064*	
C2	0.9708 (7)	0.8304 (5)	0.08140 (11)	0.0285 (9)	
C5	0.7006 (8)	0.7604 (6)	0.14990 (12)	0.0345 (10)	
H5A	0.6507	0.7108	0.1767	0.041*	
H5B	0.578	0.8286	0.1377	0.041*	
C13	0.4867 (8)	0.2558 (6)	0.18589 (14)	0.0396 (10)	
H13	0.3523	0.2426	0.1714	0.048*	
C12	0.5156 (9)	0.1720 (6)	0.22507 (14)	0.0445 (11)	
H12	0.401	0.1021	0.2363	0.053*	
C11	0.7107 (9)	0.1916 (6)	0.24725 (14)	0.0443 (12)	
H11	0.7278	0.1363	0.2735	0.053*	
C14	0.9890 (9)	1.0887 (7)	0.01517 (15)	0.0544 (13)	
H14A	1.0569	1.1193	−0.0114	0.082*	
H14B	1.0205	1.1817	0.0355	0.082*	
H14C	0.8304	1.078	0.0115	0.082*	
C7	0.3927 (9)	0.4733 (7)	0.10987 (16)	0.0524 (13)	
H7A	0.3922	0.5252	0.082	0.079*	
H7B	0.3199	0.5539	0.1293	0.079*	
H7C	0.3147	0.36	0.1094	0.079*	

### Atomic displacement parameters (Å^2^)

**Table:**

	*U*^11^	*U*^22^	*U*^33^	*U*^12^	*U*^13^	*U*^23^
S1	0.0354 (5)	0.0314 (4)	0.0300 (4)	−0.0059 (5)	0.0034 (5)	0.0035 (4)
O1	0.056 (2)	0.0317 (14)	0.0304 (13)	−0.0091 (19)	0.0042 (15)	−0.0028 (11)
N1	0.0319 (17)	0.0246 (15)	0.0334 (15)	−0.0086 (18)	0.0047 (14)	0.0014 (14)
O2	0.066 (2)	0.0325 (15)	0.0543 (17)	−0.023 (2)	0.0150 (19)	−0.0100 (13)
C9	0.034 (2)	0.0334 (19)	0.0379 (19)	−0.002 (2)	0.0054 (17)	0.0015 (18)
C1	0.030 (2)	0.0288 (18)	0.0245 (15)	−0.003 (2)	−0.0033 (16)	0.0029 (14)
C6	0.040 (3)	0.0282 (18)	0.0352 (19)	−0.010 (2)	0.0001 (19)	0.0041 (16)
C4	0.052 (2)	0.0315 (18)	0.0287 (17)	−0.007 (3)	0.009 (2)	−0.0067 (16)
C3	0.037 (2)	0.0305 (19)	0.0348 (19)	−0.003 (2)	0.0052 (19)	−0.0037 (17)
C8	0.036 (2)	0.0214 (17)	0.0341 (18)	0.0030 (19)	0.0052 (17)	−0.0042 (15)
C10	0.042 (2)	0.041 (2)	0.039 (2)	0.006 (3)	−0.001 (2)	−0.0008 (18)
C15	0.031 (2)	0.055 (3)	0.042 (2)	−0.006 (3)	0.008 (2)	0.001 (2)
C2	0.033 (2)	0.0234 (17)	0.0292 (17)	−0.0035 (18)	0.0036 (16)	0.0020 (14)
C5	0.042 (2)	0.030 (2)	0.0321 (19)	−0.002 (2)	0.0077 (19)	−0.0005 (16)
C13	0.034 (2)	0.037 (2)	0.048 (2)	−0.003 (2)	0.001 (2)	0.0091 (19)
C12	0.046 (3)	0.040 (2)	0.048 (3)	−0.006 (3)	0.012 (2)	0.013 (2)
C11	0.057 (3)	0.041 (2)	0.035 (2)	0.013 (3)	0.009 (2)	0.0050 (19)
C14	0.051 (3)	0.057 (3)	0.055 (3)	0.004 (3)	0.004 (2)	0.026 (2)
C7	0.042 (3)	0.057 (3)	0.058 (3)	−0.016 (3)	−0.015 (3)	0.019 (2)

### Geometric parameters (Å, °)

**Table:**

S1—C2	1.729 (4)	C8—C13	1.378 (6)
S1—C15	1.796 (5)	C10—C11	1.379 (7)
S1—C14	1.799 (5)	C10—H10	0.93
O1—C1	1.250 (4)	C15—H15A	0.96
N1—C1	1.364 (5)	C15—H15B	0.96
N1—C5	1.473 (5)	C15—H15C	0.96
N1—C6	1.479 (5)	C5—H5A	0.97
O2—C3	1.244 (5)	C5—H5B	0.97
C9—C10	1.391 (6)	C13—C12	1.395 (6)
C9—C8	1.402 (6)	C13—H13	0.93
C9—H9	0.93	C12—C11	1.369 (7)
C1—C2	1.455 (5)	C12—H12	0.93
C6—C7	1.531 (7)	C11—H11	0.93
C6—C8	1.538 (5)	C14—H14A	0.96
C6—H6	0.98	C14—H14B	0.96
C4—C5	1.510 (6)	C14—H14C	0.96
C4—C3	1.513 (5)	C7—H7A	0.96
C4—H4A	0.97	C7—H7B	0.96
C4—H4B	0.97	C7—H7C	0.96
C3—C2	1.415 (5)		
			
C2—S1—C15	107.6 (2)	H15A—C15—H15B	109.5
C2—S1—C14	107.0 (2)	S1—C15—H15C	109.5
C15—S1—C14	99.9 (2)	H15A—C15—H15C	109.5
C1—N1—C5	119.4 (3)	H15B—C15—H15C	109.5
C1—N1—C6	119.0 (3)	C3—C2—C1	124.4 (3)
C5—N1—C6	117.5 (3)	C3—C2—S1	120.1 (3)
C10—C9—C8	120.6 (4)	C1—C2—S1	114.9 (3)
C10—C9—H9	119.7	N1—C5—C4	110.5 (3)
C8—C9—H9	119.7	N1—C5—H5A	109.5
O1—C1—N1	121.4 (3)	C4—C5—H5A	109.5
O1—C1—C2	122.4 (3)	N1—C5—H5B	109.5
N1—C1—C2	116.2 (3)	C4—C5—H5B	109.5
N1—C6—C7	110.2 (4)	H5A—C5—H5B	108.1
N1—C6—C8	111.2 (3)	C8—C13—C12	120.5 (4)
C7—C6—C8	114.1 (4)	C8—C13—H13	119.8
N1—C6—H6	107	C12—C13—H13	119.8
C7—C6—H6	107	C11—C12—C13	120.9 (4)
C8—C6—H6	107	C11—C12—H12	119.6
C5—C4—C3	110.8 (3)	C13—C12—H12	119.6
C5—C4—H4A	109.5	C12—C11—C10	119.6 (4)
C3—C4—H4A	109.5	C12—C11—H11	120.2
C5—C4—H4B	109.5	C10—C11—H11	120.2
C3—C4—H4B	109.5	S1—C14—H14A	109.5
H4A—C4—H4B	108.1	S1—C14—H14B	109.5
O2—C3—C2	124.2 (4)	H14A—C14—H14B	109.5
O2—C3—C4	120.7 (4)	S1—C14—H14C	109.5
C2—C3—C4	115.1 (3)	H14A—C14—H14C	109.5
C13—C8—C9	118.4 (4)	H14B—C14—H14C	109.5
C13—C8—C6	121.6 (4)	C6—C7—H7A	109.5
C9—C8—C6	119.9 (4)	C6—C7—H7B	109.5
C11—C10—C9	120.1 (5)	H7A—C7—H7B	109.5
C11—C10—H10	120	C6—C7—H7C	109.5
C9—C10—H10	120	H7A—C7—H7C	109.5
S1—C15—H15A	109.5	H7B—C7—H7C	109.5
S1—C15—H15B	109.5		
			
C5—N1—C1—O1	−164.3 (4)	O2—C3—C2—S1	5.4 (7)
C6—N1—C1—O1	−7.7 (6)	C4—C3—C2—S1	−172.1 (3)
C5—N1—C1—C2	15.5 (5)	O1—C1—C2—C3	−169.5 (4)
C6—N1—C1—C2	172.0 (3)	N1—C1—C2—C3	10.7 (6)
C1—N1—C6—C7	−90.2 (5)	O1—C1—C2—S1	1.8 (5)
C5—N1—C6—C7	66.8 (5)	N1—C1—C2—S1	−178.0 (3)
C1—N1—C6—C8	142.2 (4)	C15—S1—C2—C3	46.9 (4)
C5—N1—C6—C8	−60.8 (5)	C14—S1—C2—C3	−59.6 (4)
C5—C4—C3—O2	151.0 (4)	C15—S1—C2—C1	−124.8 (3)
C5—C4—C3—C2	−31.4 (6)	C14—S1—C2—C1	128.7 (3)
C10—C9—C8—C13	−0.6 (6)	C1—N1—C5—C4	−48.4 (5)
C10—C9—C8—C6	−177.2 (4)	C6—N1—C5—C4	154.7 (3)
N1—C6—C8—C13	149.8 (4)	C3—C4—C5—N1	54.5 (5)
C7—C6—C8—C13	24.4 (6)	C9—C8—C13—C12	−0.3 (6)
N1—C6—C8—C9	−33.6 (5)	C6—C8—C13—C12	176.3 (4)
C7—C6—C8—C9	−159.1 (4)	C8—C13—C12—C11	0.9 (7)
C8—C9—C10—C11	0.8 (6)	C13—C12—C11—C10	−0.7 (7)
O2—C3—C2—C1	176.3 (4)	C9—C10—C11—C12	−0.2 (7)
C4—C3—C2—C1	−1.2 (6)		

### Hydrogen-bond geometry (Å, °)

**Table:**

*D*—H···*A*	*D*—H	H···*A*	*D*···*A*	*D*—H···*A*
C7—H7C···O2^i^	0.96	2.36	3.315 (6)	172
C15—H15A···O1^ii^	0.96	2.38	3.167 (5)	138

Symmetry codes: (i) *x*−1, *y*−1, *z*; (ii) *x*+1/2, −*y*+3/2, −*z*.
